# Supplementary material for: Preserving a qubit during state-destroying operations on an adjacent qubit at a few micrometers distance
Source: Nat Commun. 2024 Aug 3;15:6575. doi: 10.1038/s41467-024-50864-2 (PMC11298003; doi:10.1038/s41467-024-50864-2)
Supplement: Supplementary file 1 — Supplementary Information [file 41467_2024_50864_MOESM1_ESM.pdf]

Supplementary Information for “Preserving a qubit during state-destroying operations on an adjacent qubit at a few micrometers distance”

Sainath Motlakunta[1,2,*] Nikhil Kotibhaskar[1,2], Chung-You Shih[1,2], Anthony Vogliano[1,2], Darian Mclaren[1,2], Lewis Hahn[1,2], Jingwen Zhu[1,2], Roland Hablützel[1,2], and Rajibul Islam[1,2]

[1] *Institute for Quantum Computing, University of Waterloo, Waterloo, Ontario N2L 3G1, Canada and*
[2] *Department of Physics and Astronomy, University of Waterloo, Waterloo, Ontario N2L 3G1, Canada*

(Dated: July 23, 2024)

Supplementary Note 1. DETAILED EXPERIMENTAL SETUP

Our apparatus (Supplementary Fig. 1) consists of ^{171}Yb^{+} ions trapped in a four-rod paul trap with radial secular frequencies (ω_x,ω_z) of around $2\pi\times$ 1.1 MHz and axial trap frequencies (ω_y) of $2\pi\times$ 270 kHz. The ground state hyperfine levels $S_{1/2}\,|F=0,m_F=0\rangle$ and $S_{1/2}\,|F=1,m_F=0\rangle$ (separated by 12.642813 GHz) are assigned as the $|{\downarrow}\rangle$ and $|{\uparrow}\rangle$ of effective spin-1/2 particle, respectively. A magnetic field (B) perpendicular to the ion chain provides the quantization axis and a Zeeman splitting of $\Delta_{\rm zm} = 2\pi\times 3.25$ MHz between $S_{1/2}\,|F=1,m_F=0\rangle$ and $S_{1/2}\,|F=1,m_F=1\rangle$ levels. Global Doppler cooling, state-detection, and optical pumping all derived from a laser source (369nm-laser-1) along with repump beams (935 nm) are illuminated onto the ions in the XY plane. A probe beam (along z) is illuminated onto the ions through an addressing system of effective numerical aperture(NA) of 0.16(1). This probe beam is resonant to $D_1^{(10)}$ or $D_1^{(11)}$ transitions to perform site-selective state reset or measurement. We use another 369nm(369nm-laser-2) source for the probe beams whose frequency can be independently tuned (without affecting the global detection and cooling beams) to either $D_1^{(10)}$ or $D_1^{(11)}$ transitions. The fluorescence from the ions along z is collected through an in-house built objective onto a PMT(Hamamatsu: H10682-210) through a pellicle beam splitter(45:55) (Thorlabs: BP145B5). The optical access to the ion in our apparatus, perpendicular to the probe beam direction (z), is limited to an NA of less than 0.1. Due to this limitation of our ion trap apparatus, we are unable to perform high-fidelity state detection of the ions while they are being probed using the state-detection beam.

The state-dependent fluorescence transmitted after the trap is also monitored using a CMOS camera C2 (FLIR: Blackfly S BFS-PGE-04S2M) as shown in Supplementary Fig. 1. A microwave field drives the $|{\downarrow}\rangle$ to $|{\uparrow}\rangle$ transition. An acoustic-optic modulator (AOM1) in a double pass configuration, placed after the 369nm-laser-2, is used as a switch with precise timing and power control for the probe light. The light is then coupled to a PM fiber which is then expanded using a single lens(L1) and is polarization-cleaned using a polarizer. The light is sampled onto a photodiode (PD) that is used to stabilize the intensity fluctuations using PID feedback to the AOM. The polarization-cleaned and power-stabilized light from the PM fiber illuminates a Digital Micromirror device (DMD) (Visitech Luxbeam 4600 DLP) placed in the Fourier plane. A motorized $\lambda/2$ waveplate(WP1) is placed after the DMD to control the final polarization of the light. The DMD is programmed with an aberration-corrected amplitude hologram generated from an iterative Fourier transform algorithm (IFTA)(Supplementary Note 8) to produce a Gaussian beam of waist w=1.50(5) μm in the ion plane (IP2). The negative first-order beam diffracted from the hologram on DMD is then relayed to the ion through the reflection of the pellicle. A flip mirror placed before the intermediate image plane IP1 is used to image the IP1 onto a camera C1 for initial characterization. Due to the limitations of our trap parameters, such as maximum electrode voltage, we could trap two ^{171}Yb^{+} ions with an inter-ion spacing no smaller than 9 μm $= 6w$.

Despite designating the ground state hyperfine levels of ^{171}Yb^{+} ions ($S_{1/2}\,|F=0,m_F=0\rangle$ and $S_{1/2}\,|F=1,m_F=0\rangle$) with $|{\downarrow}\rangle$ and $|{\uparrow}\rangle$ of an effective spin-1/2 particle, the measurement and reset processes involve additional states. Consequently, the ions may ultimately occupy the states $S_{1/2}\,|F=1,m_F=-1\rangle$ and $S_{1/2}\,|F=1,m_F=1\rangle$ outside the Hilbert space of the qubit. To model the dynamics of the ion pertaining to this work, we account for eight levels in our Hilbert space, 4 for $S_{1/2}$ and 4 for $P_{1/2}$ (Supplementary Fig. 2). The process of state detection mixes the ion in state $|2\rangle$ with states $|1\rangle$ and $|3\rangle$, and with $|0\rangle$ when off-resonant excitation to $P_{1/2}\,|F=1\rangle$ states are included. The process of state reset mixes the ion in state $|2\rangle$ with states $|0\rangle$, $|1\rangle$ and $|3\rangle$. The microwave field used in

* smotlaku@uwaterloo.ca

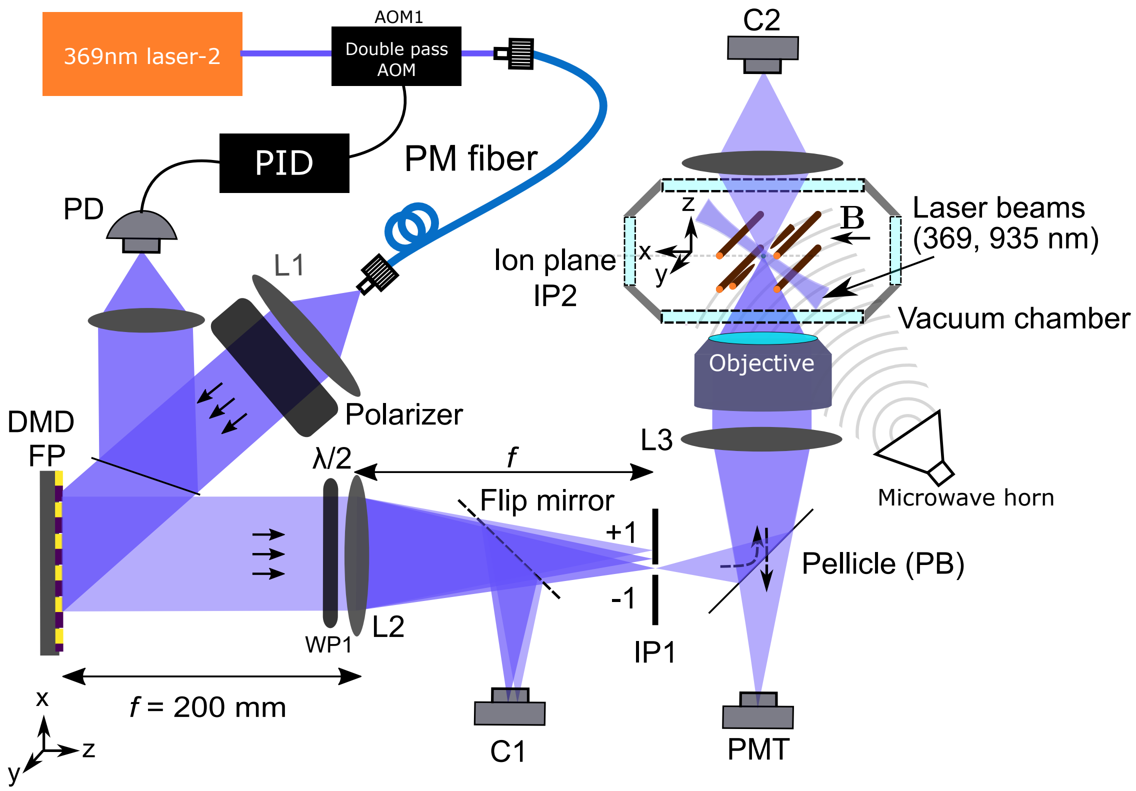

Supplementary Fig. 1. Experimental setup

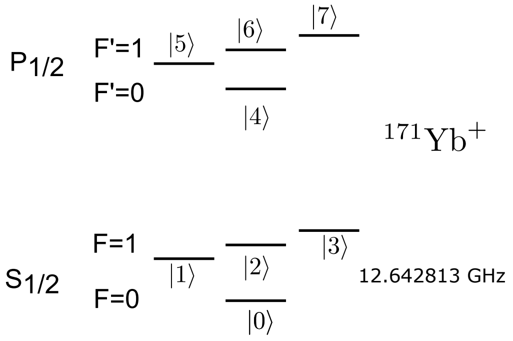

Supplementary Fig. 2. Encoding for $S_{1/2}$ and $P_{1/2}$ energy levels of $^{171}\mathrm{Yb}^+$ ion

the Ramsey measurements (Supplementary Note 2) only couples the levels $|2\rangle$ and $|0\rangle$.

Supplementary Note 2. RAMSEY INTERFEROMETRY

To estimate the Fidelity $F_{1|2}$ (main text Eq. 1), we use a set of Ramsey measurements to characterize the P_{AQM} caused by the probe beam parked at a distance d from the ion-1. (Main text Fig. 2). Each set of measurements is initialized by a sequence of Doppler cooling for 2.5 ms, optically pump(global) to $|\downarrow\rangle$ ($|0\rangle$) for 20 μs. The probe light is illuminated for a time T between two microwave $\pi/2$ pulses (detuned from a transition $|\downarrow\rangle$ to $|\uparrow\rangle$ by $\Delta_{\mu\mathrm{w}} = 2\pi \times 10$ kHz) for a duration of about 6 μs each. Here, $\Delta_{\mu\mathrm{w}}$ is chosen such that the time periods of the Ramsey oscillations are much smaller than the characteristic decay times (T_2^*), while ensuring that $\Delta_{\mu\mathrm{w}}$ is smaller than the microwave-induced Rabi oscillation frequency of 100 kHz. A detection step follows where the ions are illuminated by a global detection beam for 1.5 ms, during which the state-dependent fluorescence from the ions is collected using a PMT. Each such experiment is repeated 200 times, and the PMT counts are averaged over. The averaged PMT counts are then normalized using measured counts from preparing $|\downarrow\rangle$ and $|\uparrow\rangle$ states. The normalized fluorescence($\approx P(|\uparrow\rangle)$) oscillates at a frequency of 10 kHz as the time T is varied. We denote the contrast of these oscillations by $R_c(T)$. To extract the characteristic decay time(T_2^*) of the Ramsey contrast $R_c(T)$ for a given configuration of d, these Ramsey measurements are done with varying T (main text Fig. 2a). Using the preliminary coarse estimate of Ramsey contrast, T_2^* is roughly estimated, and the time interval between 10µs and $2T_2^*$ is divided into

five intervals, with each interval containing 21 data points in a span of 200 µs.

After the Ramsey measurements for these five intervals, the PMT counts are fit using the following function to extract the T_2^*.

$$f(T)_{T_2^*,\alpha,\beta,\phi,C} = \sin^2(\omega T + \phi)(\alpha e^{-T/T_2^*}) + \beta(1 - e^{-T/T_2^*}) + C \tag{S1}$$

As a baseline measurement, we characterize the Ramsey measurements with no probe beam during the wait time (Supplementary Fig. 3) and estimate that the T_2^* is much larger than 200ms. This large T_2^* corresponds to an infidelity (1-$F_{1|2}$) $< 3 \times 10^{-5}$.

Supplementary Note 3. FIDELITY ESTIMATION

To quantify how well the quantum state of ion-1 is preserved after an operation on ion-2, we use the fidelity metric ($F_{1|2}$) [1] defined as

$$F_{1|2}(t) = \mathrm{tr}\left(\sqrt{\rho(0)^{1/2}\rho(t)\rho(0)^{1/2}}\right) \tag{S2}$$

where $\rho(0)$ and $\rho(t)$ denote density matrix operators of ion-1 (assuming unentangled with ion-2) before and after a state-reset or measurement operation (performed for time t) on ion-2, respectively. This metric yields a different value based on the initial state of ion-1, and using numerical simulations (Supplementary Note 5), we find that $\rho(0) = |2\rangle\langle 2|$ represents the worst case scenario (Supplementary Fig. 4).

By analytically solving the master equation of the system we find that the Ramsey fringe contrast $R_c(T)$ could be used to estimate the worst-case fidelity of ion-1 after an operation ion-2 for a time (T) using

$$F_{1|2}(T) = \frac{2}{3}R_c(T) + \frac{1}{3} \tag{S3}$$

To derive the above expression analytically, we assume that the intensity of probe light decohering the ion-1 is very weak, that it causes a low probability of accidental measurement $P_{\mathrm{AQM}} << 1$. In this limit, consider the density matrix of ion-1 in a reduced Hilbert space with only ($|0\rangle, |1\rangle, |2\rangle, |3\rangle$) states. We model the action of probe light using the collapse operators $C_n = \sqrt{\gamma_n}\,|i\rangle\langle j|$ where $i, j \in \{0, 1, 2, 3\}$ where γ_n is the rate of collapse. The collapse operators and their rates depend on the transition the probe is driving and its polarization. For example, in state detection, only the probe with π polarization causes the AQM of ion-1. In the limit $P_{\mathrm{AQM}} << 1$ with $\gamma << 1$ representing the rate of P_{AQM} we use the the following collapse operators

Transition	Polarization	Collapse operators
$D_1^{(10)}$	π	$\sqrt{\frac{\gamma}{3}}\,|2\rangle\langle 2|$, $\sqrt{\frac{\gamma}{3}}\,|1\rangle\langle 2|$, $\sqrt{\frac{\gamma}{3}}\,|3\rangle\langle 2|$
$D_1^{(11)}$	σ^+	$\sqrt{\frac{\gamma}{3}}\,|2\rangle\langle 2|$, $\sqrt{\frac{\gamma}{3}}\,|3\rangle\langle 2|$, $\sqrt{\frac{\gamma}{3}}\,|0\rangle\langle 2|$
$D_1^{(11)}$	σ^-	$\sqrt{\frac{\gamma}{3}}\,|2\rangle\langle 2|$, $\sqrt{\frac{\gamma}{3}}\,|1\rangle\langle 2|$, $\sqrt{\frac{\gamma}{3}}\,|0\rangle\langle 2|$

For ion-1 initialized in $\rho(0) = |2\rangle\langle 2|$ state, the final state of the ion-1 after the AQM due to weak probe for a time t is calculated by analytically solving the Lindblad master equation

$$\dot{\rho}(t) = -\frac{i}{\hbar}[H_{atom}, \rho(t)] + \sum_n \frac{1}{2}\left[2C_n\rho(t)C_n^\dagger - \rho(t)C_n^\dagger C_n - C_n^\dagger C_n\rho(t)\right] \tag{S4}$$

Here, H_{atom} in interaction picture is given by

$$H_{atom} = -(\Delta_{\mu\mathrm{w}} + \Delta_{\mathrm{zm}})\,|1\rangle\langle 1| - \Delta_{\mu\mathrm{w}}\,|2\rangle\langle 2| - (\Delta_{\mu\mathrm{w}} - \Delta_{\mathrm{zm}})\,|3\rangle\langle 3|$$

Here $\Delta_{\mu\mathrm{w}}, \Delta_{\mathrm{zm}}$ denote the detuning of the microwave field and Zeeman splitting, respectively. From the solution, we find the fidelity $F_{1|2}$ to be

$$F_{1|2}(t) = \sqrt{\rho_{22}(t)} = e^{-\frac{1}{3}\gamma t} \approx 1 - \frac{1}{3}\gamma t \tag{S5}$$

Similarly, after the Ramsey experiment (Supplementary Note 2), the normalized is given as

$$\rho_{22}(t) = \frac{1}{4} + \frac{1}{4}e^{-1/2\gamma t - i\Delta_{\mu\mathrm{w}}t} + \frac{1}{4}e^{-1/2\gamma t + i\Delta_{\mu\mathrm{w}}t} + \frac{1}{4}e^{-2/3\gamma t} \tag{S6}$$

The Ramsey fringe contrast is then given by $R_c(T) = \rho_{22}\left(\frac{(2m+1)\pi}{\Delta_{\mu\mathrm{w}}}\right) - \rho_{22}\left(\frac{2m\pi}{\Delta_{\mu\mathrm{w}}}\right)$ for a positive integer m and assuming $\gamma << \Delta_{\mu\mathrm{w}}$ we get

$$R_c(T) \approx e^{-\frac{1}{2}\gamma t} \approx 1 - \frac{1}{2}\gamma t \tag{S7}$$

Combining Eq. S5 and S7 we get S3. Further, the Ramsey fringe decays exponentially with a characteristic time T_2^* which leads to

$$F_{1|2}(T) = \frac{2}{3}e^{-(T/T_2^*)} + \frac{1}{3} \tag{S8}$$

Supplementary Note 4. CALIBRATIONS

A. FP aberration phase profile calibration

We characterize optical aberrations in the entire beam path in terms of a Fourier plane (FP) phase map. The optical aberrations till IP1 ($\Phi_{ab}^{(0)} + \Phi_{ab}^{(1)}$)

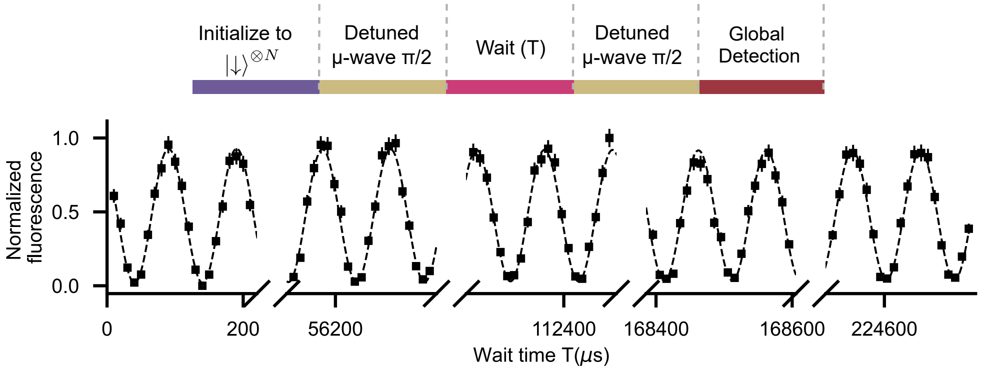

Supplementary Fig. 3. **Ramsey measurements without probe beam**
(Top) Ramsey interferometric protocol to measure qubit coherence time T_2^* without the probe beam applied. (Bottom) Ramsey fringes in normalized fluorescence originating from the detuning between the microwave source and the qubit frequency. Here, the background-subtracted fluorescence counts are measured during the global detection step and are normalized with respect to the counts from $|\uparrow\rangle$. Error bars indicate standard error from 200 experimental repetitions.

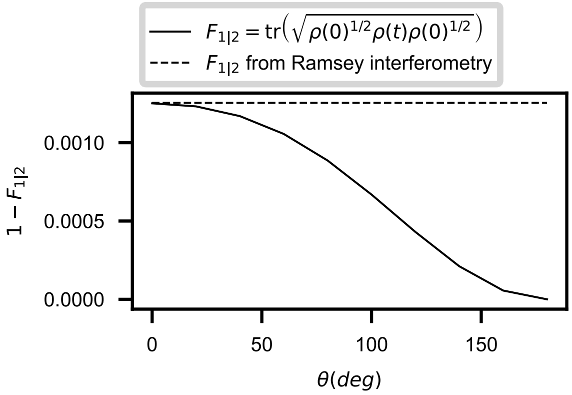

Supplementary Fig. 4. **Infidelity(1-$F_{1|2}$) as a function of Bloch angle** θ. Here the ion-1 is initialized in state $\rho(0) = |\psi\rangle\langle\psi|$ where $|\psi\rangle = cos(\theta/2)|2\rangle + sin(\theta/2)|0\rangle$. The $F_{1|2}$ (Eq. S2) is measured after a weak state-detection $(D_1^{(10)})$ light of intensity $I = 5 \times 10^{-5} I_{\text{sat}}$ and polarization $I_\pi/I = \frac{1}{3}$ is applied on ion-1 for 11μs. For comparison, the infidelity $(1 - F_{1|2})$is shown(dotted line) from Ramsey interferometry(Supplementary Note 2) where a probe light of similar parameters as above is illuminated on ion-1 during the wait time.

are characterized using the camera C1 [2] as a sensor to measure the relative optical phase between two FP ‘patches’(Supplementary Fig. 5a). The optical aberrations from IP1 to IP2 $(\Phi_{ab}^{(2)})$ are measured using a single ion as a sensor (see main text methods)(Supplementary Fig. 5b). The phase profile $\Phi_{ab}^{(0)} + \Phi_{ab}^{(1)} + \Phi_{ab}^{(2)}$ is used to compensate for optical aberrations using an iterative Fourier transform algorithm (IFTA) [2] to create a diffraction-limited gaussian beam spot in IP2.

B. Fourier plane intensity profile calibration

The incident light on DMD from L1 is nonuniform and has a Gaussian intensity profile. Further, the pellicle beam splitter (PB) has an angular dependence on reflection. The effective intensity profile on the FP is measured using an ion in IP2 as a sensor. The ion is prepared in $|2\rangle$ state, and the optical pumping light from DMD is used to pump to $|0\rangle$ state for a fixed time. The value of the intensity of probe light reflected by a circular patch(30 pixels diameter) on DMD is inferred from the decrease in ion fluorescence. This measurement is repeated for different phase-corrected (Supplementary Note 4 A) patches on the DMD to construct an effective intensity profile. The intensity profile is then smoothened and interpolated, and a square root of the intensity profile is used as the amplitude profile of the incident electric field. This amplitude profile is further used as an input to IFTA hologram generation algorithm [2].

C. Relative Intensity calibration

The intensity of the probe light illuminating the ion through DMD is controlled by adjusting the RF power of the AOM1(Supplementary Fig. 1). The RF power vs. intensity of the light is calibrated using camera C1. To ensure accurate reporting, the linearity of C1’s exposure time is confirmed over four orders of magnitude from 100 μs to 5 s (Supplemen-

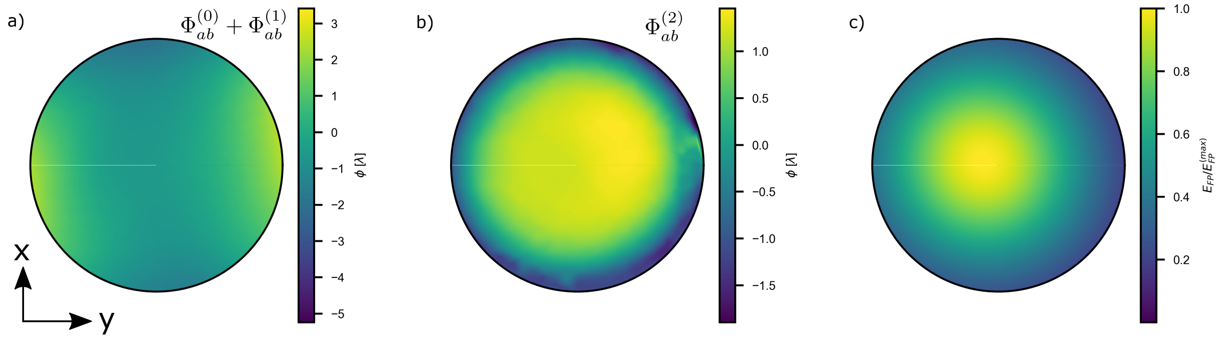

Supplementary Fig. 5. **FP phase and amplitude profile**. a)The aberration phase profile was measured using camera C1. b) The aberration phase profile is measured using the ion at IP2. For a-b, the piston and tilt terms are removed from the measured phase profiles, and the profile is further smoothened and interpolated. c) The scaled amplitude profile measured at IP2. The measured amplitude profile is smoothened, interpolated, and fit to 2D Gaussian.

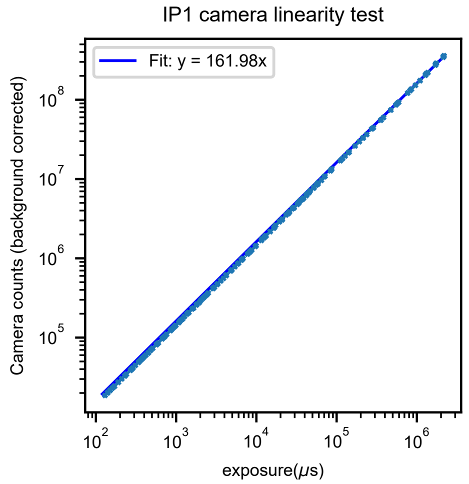

Supplementary Fig. 6. **C1 camera linearity calibration**. When a probe beam of a given intensity illuminates the camera, the data represents the counts from the camera, corrected for background, as the camera's exposure is increased. Since the camera does not have a large dynamic range, the intensity of the incident probe beam is varied by a known amount, followed by the measurements taken within the dynamic range of the camera. All the data is then stitched together to obtain the plot above. Error bars represent the standard error from 5 measurement repetitions.

tary Fig. 6). The linearity of the RF source power setting and the RF power output is calibrated using a spectrum analyzer. The pellicle placed after the IP1 has a polarization-dependent transmission profile that is calibrated using camera C2 and compensated using the AOM. This calibration also gives a relative measurement connecting the attenuated and unattenuated probe beam intensity(main text Fig. 3b and Fig. 4b) through the camera's exposure time and pixel intensity (at a fixed gain).

D. Absolute intensity and polarization calibration

The intensity of the probe beam (calibrated using C1) on the ion is calibrated with respect to the saturation intensity (I_{sat}) of the ion. A series of optical pumping experiments are done with the probe using varying calibrated power and input polarization(varied using the $\lambda/2$ waveplate WP). These experiments are then fit using numerical simulations to extract the absolute intensity and polarization of the light illuminating the ion.

E. Probe beam position and size calibration

The position of the probe beam and its beam waist is calibrated by using a single ion as a sensor for the intensity. The ion is initialized in state $|2\rangle$, and the probe beam (state-reset) illuminates the ion for a time smaller than the optical pumping time, followed by a state measurement. The dependence of ion fluorescence as a function of beam position is used to extract the relative beam position and the beam waist (Supplementary Fig. 7). Here the position of the probe beam is changed by programming the hologram on the Digital micromirror device (DMD) to

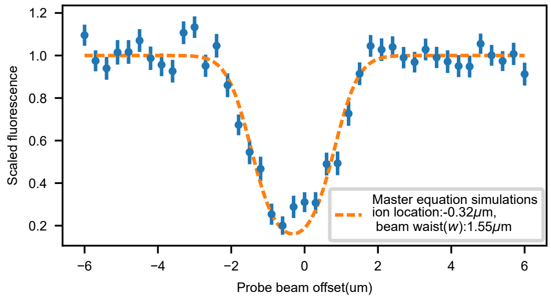

Supplementary Fig. 7. **Probe beam position and size calibration**. The data represents state-detection fluorescence counts from a single ion after it is optically pumped using a state-reset probe beam with a position offset. Error bars indicate the standard error from 200 experimental repetitions. The dashed orange line represents the master equation simulation of a similar sequence, simulating the ion's state when a state-reset probe beam with a beam waist of $w = 1.55$ µm and a position offset of -0.32 µm is applied.

generate a shifted Gaussian beam. This procedure is regularly done before every set of experiments to fix the slow drift of the relative position of the beam to the ion. We measured that the probe beam drifts by about 0.20(15) µm over the period of 15 min (a single Ramsey measurement).

F. Length scale calibration

The imaging system's effective focal length ($\approx$ 24mm) translates the known length scale in the Fourier plane (FP) to the length scale in IP2. We find the relative beam positions of two ions in a trap using an experiment similar to (Supplementary Note 4 E). The inter-ion spacing could be calculated from the difference between the estimated relative beam positions of the two ions. This estimated inter-ion spacing is compared to an estimation of equilibrium positions (estimated from the measured trap frequencies) to further calibrate the system's effective focal length. We could calibrate the length scale in IP2 using this method to within 5% accuracy.

G. Frequency calibration

The relative shift of the laser frequency is calibrated by tuning the laser to the optical pumping transition and maximizing its pumping efficiency onto the ion.

Supplementary Note 5. SETUP FOR NUMERICAL SIMULATIONS OF LINDBLAD MASTER EQUATION

To model the dynamics of the ion pertaining to this work, the relevant levels are within the $S_{1/2}$ and the $P_{1/2}$ manifolds (Supplementary Fig. 2) The Hamiltonian, describing the interaction with radiation, accounts for couplings due to optical pumping, state detection, and the microwave. For the purpose of efficient numerical simulations, it is useful to remove the time dependence through a rotating transform $(U(t))$ [3] such that $H_{rot} = UHU^\dagger - U\frac{d}{dt}U^\dagger$. We find that when the optical pumping, detection, and microwave couplings are monochromatic, the solution of U exists, and we use this to remove the time dependence from our total Hamiltonian. With the time dependence of the Hamiltonian accounted for, the time evolution of the density matrix can be determined by solving the Lindblad master equation(Eq. S4) with appropriate collapse operators due to the spontaneous emission. Using such numerical simulations, the evolution of the density matrix is calculated in a Ramsey interferometry(Supplementary Note 2). T_2^* is extracted from the simulations, and the dependence as a function of input intensity on ion-1 is calculated. This dependence is used to extract the intensity crosstalk I_X from measured T_2^* of Ramsey measurements.

A. Rabi fequencies

The rabi frequencies for a simple 2-level system are set according to the formula:

$$\frac{I}{I_{\text{sat}}} = \frac{2\Omega^2}{\Gamma^2},$$

where I is the intensity of the laser, I_{sat} is the saturation intensity, and Γ is the spontaneous emission rate of the transition. In our case, we are interested in finding the rabi frequency pertaining to a specific transition i.e

$$\Omega_{F,m_F,F',m_{F'}} = \frac{\langle F, m_F|d \cdot E|F', m_{F'}\rangle}{\hbar}$$

Applying the Wigner-Eckart theorem, we get

$$\langle F, m_F|d_q|F', m_{F'}\rangle = \langle F, m_F|F', m_{F'}, 1, q\rangle \; \langle F||d_q||F'\rangle$$

Now, the reduced matrix element can be further broken down as

$$\langle F||d||F'\rangle = \langle JIF||d||J'IF'\rangle = \langle J||d||J'\rangle (-1)^{F'+J+1+I} \sqrt{(2F'+1)(2J+1)} \begin{Bmatrix} J & J' & 1 \\ F' & F & I \end{Bmatrix}$$

The reduced matrix element between the J levels can simply be calculated from the decay rate of the excited state using Fermi's golden rule as follows:

$$\Gamma_{JgJe} = \frac{\omega_0^3}{3\pi\epsilon_0\hbar c^3}\frac{2J_g+1}{2J_e+1}|\langle J_g||d||J_e\rangle|^2$$

Since it is commonly used in $^{171}\mathrm{Yb}^+$ literature, we introduce the saturation intensity as defined for the $^2\mathrm{S}_{1/2}$ to $^2\mathrm{P}_{1/2}$ ignoring the internal structure:

$$I_{\mathrm{sat}} = \frac{\pi\Gamma ch}{3\lambda^3}$$

Combining these equations along with $|E| = \sqrt{I/2c\epsilon_0}$ we get

$$\Omega^2_{F,m_F,F',m_{F'}} = \frac{I}{I_{\mathrm{sat}}}\frac{\Gamma^2_{JgJe}}{2}|\langle F,m_F|F',m_{F',1,q}\rangle|^2 \times (2F'+1)(2J+1)\begin{Bmatrix} J & J' & 1 \\ F' & F & I \end{Bmatrix}^2 \times \frac{2J_e+1}{2J_g+1}$$

In the case of $^{171}\mathrm{Yb}^+$, for all the allowed transitions between $^2\mathrm{S}_{1/2}$ and $^2\mathrm{P}_{1/2}$, the second line of the above expression evaluates to 1/3, leaving us with a particularly simple expression for the rabi frequency

$$\Omega^2 = \frac{I}{I_{\mathrm{sat}}}\frac{\Gamma^2}{6} \tag{S9}$$

Supplementary Note 6. PROCESS QUBIT DETECTION EFFICIENCY

Consider a probe beam resonant to $D_1^{(10)}$ transition illuminates the process qubit for a time t. The scattered light from the process qubit is collected using a detector of efficiency $\varepsilon_{\mathrm{sys}}$. The state of the qubit is inferred to be $|\uparrow\rangle$ if the detector registers a single count. We use the approach presented in Ref. [4–6] to estimate the detection fidelity. The error in detecting the $|\uparrow\rangle$ is given by the probability of detecting no photons when the qubit is initialized in $|\uparrow\rangle$ state [6].

$$P_{t,|\uparrow\rangle}(n=0) = \frac{R_{\mathrm{d}}}{\varepsilon_{\mathrm{sys}}R_{\mathrm{o}}+R_{\mathrm{d}}}e^{-R_{\mathrm{bg}}t}\left[1-e^{-(\varepsilon_{\mathrm{sys}}R_{\mathrm{o}}+R_{\mathrm{d}})t}\right] + e^{-R_{\mathrm{d}}t}e^{-((\varepsilon_{\mathrm{sys}}R_{\mathrm{o}}+R_{\mathrm{bg}})t} \tag{S10}$$

Here R_o denotes the scattering rate of state $|\uparrow\rangle$, R_b denotes the bright pumping rate, and R_d denotes the dark pumping rate [4]. We note a plausible typo in the above expression in Ref. [6] with a prefactor $\varepsilon_{\mathrm{sys}}$ missing in the exponential of the second term. Similarly, the probability of detecting no photons when the qubit is initialized in state $|\downarrow\rangle$ [6]

$$P_{t,|\downarrow\rangle}(n=0) = \frac{R_{\mathrm{b}}}{\varepsilon_{\mathrm{sys}}R_{\mathrm{o}}-R_{\mathrm{b}}}e^{-R_{\mathrm{bg}}t}\left[e^{-R_{\mathrm{b}}t}-e^{-\varepsilon_{\mathrm{sys}}R_{\mathrm{o}}t}\right] + e^{-R_{\mathrm{b}}t}e^{-R_{\mathrm{bg}}t} \tag{S11}$$

The average fidelity of state-detection of the process qubit is given by

$$F = \frac{\big(1-P_{t,|\uparrow\rangle}(n=0)\big)+P_{t,|\downarrow\rangle}(n=0)}{2}$$

Supplementary Note 7. ERROR ANALYSIS

We use the bootstrapping method to get the standard error of the fitted parameters of the population transfer or the decoherence time. We randomly resampled the dataset of the same DMD probing duration with replacement. The resampled dataset is used for extracting the fitted parameters. Repeating the resampling and fit process creates the empirical distribution of the fitting parameters. In this work, we repeatedly resample the dataset 20 times, and the standard deviation of the 20 fitting parameters of the resampled datasets is used as the error. With the error of the τ(ion2) and T_2^*(ion1) time through bootstrapping, the error of estimating fidelity can be derived with error propagation.

Supplementary Note 8. ALGORITHM FOR HOLOGRAM GENERATION

In this work, we used an iterative Fourier transform algorithm [2] to calculate the required hologram to display on the DMD. However, we improved the power efficiency(see 'power efficiency' of supplementary information in Ref. [2]) by scaling up the target field with $\frac{4}{\pi}$ during the IFTA binarization process.

This new improvement is based on the fact that for a square wave $x(t)$ its fundamental mode $\sin(\omega t)$ can have a coefficient greater than 1.

$$x(t) = \frac{4}{\pi}\sum_{k=1}^{\infty}\frac{\sin((2k-1)\omega t)}{2k-1} \tag{S12}$$

$$= \frac{4}{\pi}\left(\sin(\omega t)+\frac{1}{3}\sin(3\omega t)+\frac{1}{5}\sin(5\omega t)+...\right) \tag{S13}$$

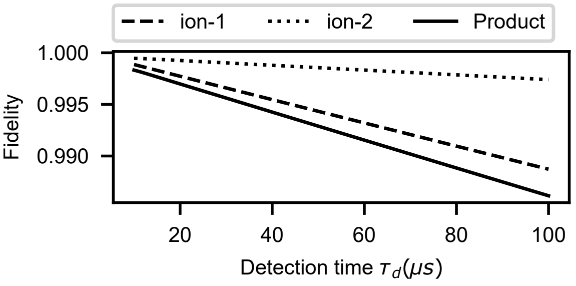

Supplementary Fig. 8. Calculated process qubit (ion2) detection fidelity[5, 6] and asset qubit (ion1) preservation fidelity ($F_{1|2}$) as a function of τ_{d} (detection time).

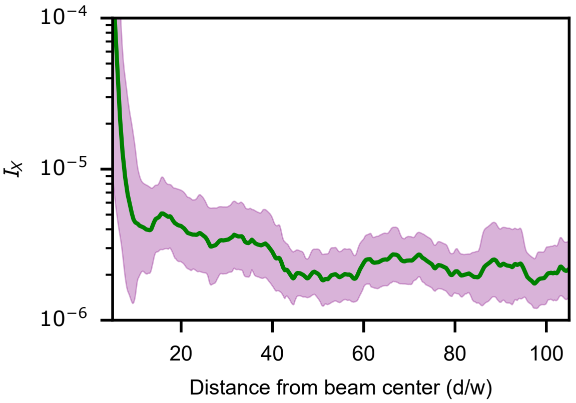

Supplementary Fig. 9. Intensity crosstalk (I_X) as a function of distance from the beam at the intermediate image plane, captured by camera C1. The green line represents a moving average with a window size of $9w$, and the purple shaded region denotes the range within one standard deviation from the moving average. This beam profile, a Gaussian beam with a waist of $w = 10$µm, was generated using the IFTA algorithm [2] with a signal window of 3mm x 0.5mm, iterated 10,000 times.

Even though DMD can have binarized control on the grating amplitude (0 1), a higher modulation level can be achieved. With the new improvement, the power of the signal can be enhanced by $(\frac{4}{\pi})^2 \approx 1.6$ times, which also effectively improves the signal-to-noise background ratio.

Supplementary Note 9. FIDELITY CALCULATIONS FOR LONG DETECTION TIMES

Figure 8 shows the estimated asset qubit and process qubit fidelities as a function of detection time. The conditions for this figure are similar to those of the main text Fig. 4d, but for longer detection times.

Supplementary Note 10. INTENSITY CROSSTALK AT THE INTERMEDIATE IMAGE PLANE

In this section, we investigate the intensity distribution of an addressing beam in the intermediate image plane. To measure the intensity crosstalk performance in the intermediate image plane (with NA $\approx$ 0.02), we generate a Gaussian beam with a beam waist ($w = 10\,\mu$m) and capture high dynamic range (HDR) images using camera C1. Figure 9 illustrates the intensity crosstalk I_X at distances ranging from $4w$ to $100w$ from the beam center. We observe that the intensity crosstalk beyond several beam waists (>$20w$) is further suppressed to below 3×10^{-6}. If this intermediate image plane distribution is relayed onto the ion, this residual intensity crosstalk corresponds to an infidelity (1-$F_{1|2}$) of less than 3×10^{-5} for state-reset under conditions similar to those presented in the main text Fig. 3c, and less than 1×10^{-4} for state-measurement of the process qubit under similar conditions as presented in the main text Fig. 4b.

The performance at the ion plane, as presented in Figs. 3c and 4b of the manuscript, shows degradation from the measured values at the intermediate image plane. We attribute this deterioration to specular reflections from the chamber windows. Another possible source of error is uncompensated higher-order aberrations. Both sources of error can be mitigated in future experiments by using better glass and coatings, and by higher-resolution aberration characterization using an ion.

[1] M. A. Nielsen and I. L. Chuang, *Quantum Computation and Quantum Information* (Cambridge University Press, 2012).
[2] C.-Y. Shih, S. Motlakunta, N. Kotibhaskar, M. Sajjan, R. Hablützel, and R. Islam, Reprogrammable and high-precision holographic optical addressing of trapped ions for scalable quantum control, npj Quantum Information **7**, 57 (2021).
[3] T. H. Einwohner, J. Wong, and J. C. Garrison, Analytical solutions for laser excitation of multilevel sys-

tems in the rotating-wave approximation, Physical Review A **14**, 1452 (1976).

[4] R. Noek, G. Vrijsen, D. Gaultney, E. Mount, T. Kim, P. Maunz, and J. Kim, High speed, high fidelity detection of an atomic hyperfine qubit, Optics Letters **38**, 4735 (2013).

[5] Mark Acton, *Detection and Control of Individual Trapped Ions and Neutral Atoms*, Ph.D. thesis, The University of Michigan (2008).

[6] S. Crain, C. Cahall, G. Vrijsen, E. E. Wollman, M. D. Shaw, V. B. Verma, S. W. Nam, and J. Kim, High-speed low-crosstalk detection of a 171Yb+ qubit using superconducting nanowire single photon detectors, Communications Physics **2**, 97 (2019).
